# Association between parental feeding styles, body mass index, and consumption of fruits, vegetables and processed foods with mothers´ perceptions of feeding difficulties in children

**DOI:** 10.1186/s12887-024-04657-7

**Published:** 2024-03-08

**Authors:** Nathalia Moretti Fontanezi, Priscila Maximino, Rachel Helena Vieira Machado, Gerson Ferrari, Mauro Fisberg

**Affiliations:** 1 Sciences Applied to Pediatrics Postgraduate Program, Federal University of São Paulo, Rua Botucatu, 598, Vila Clementino, São Paulo, 04023-062 SP Brazil; 2 Center for Excellence in Nutrition and Feeding Difficulties, PENSI Institute, Luis Egydio Setúbal Foundation, Sabará Children’s Hospital, São Paulo. Av. Angelica 1968, conj 71a 74, São Paulo, 01239-040 SP Brazil; 3 HCor Research Institute, Rua Abílio Soares, 250, São Paulo, São Paulo, CEP 04005909 SP Brazil; 4 Escuela de Ciencias de la Actividad Física, Universidad de Santiago de Chile (USACH), el Deporte y la Salud, Santiago, Chile; 5 Facultad de Ciencias de la Salud, Universidad Autónoma de Chile, Providencia, Chile

**Keywords:** Feeding difficulties, Parental feeding styles, Body mass index, Preschoolers, Nutrition, Healthy eating, Fruit and vegetable consumption

## Abstract

**Background:**

Feeding difficulties (FDs) are complex phenomena influenced by parental factors, feeding behaviour, and cultural factors. However, studies of the influences of these factors on FDs incidence are scarce. Thus, this study aimed to identify the associations between mothers’ perceptions of FDs in children and parental feeding styles, body mass index, and the consumption of fruits, vegetables and processed foods.

**Method:**

Two hundred and fifty-seven mothers of children aged 1 to 6 years and 11 months participated in this cross-sectional study and self-completed electronic questionnaires on sociographic variables, parental feeding styles, the consumption of fruits, vegetables and processed foods and FDs. Nutritional status was classified by body mass index (kg/m^2^).

**Results:**

The prevalence of FDs in children was 48.2%, and the mean age was 43.8 (± 17.6) months. The indulgent parental feeding style was the most common (40.1%), followed by the authoritative (31.1%), authoritarian (23.7%), and uninvolved (5.1%) styles. An indulgent parental feeding style (OR: 4.66; 95% CI: 2.20–9.85), a high body mass index (OR: 1.35; 95% CI: 1.09–1.68), and the consumption of processed foods (OR: 5.21; 95% CI: 2.85–9.53) were positively associated with increased odds of the absence of FDs in children. The associations of authoritarian and uninvolved parental feeding styles and the consumption of fruits and vegetables with FDs in children were not significant.

**Conclusion:**

This study identified multiple factors that are possibly associated with feeding behaviours in young children. However, further studies need to be undertaken to evaluate how such behaviours affect FDs.

## Background

Child feeding problems are common, and most children experience these difficulties for many reasons. These difficulties are concerning because they may persist and influence child development as well as heighten the risk for future eating problems in children and adolescents [1]. The initial age at which these problems occur is usually approximately two years [2, 3]. The prevalence of these problems is not clearly defined in the literature and varies (5.6–60%) due to differences in the methods used to assess the problem, the age of the children being studied and the location of the study [4]. Despite their prevalence, little is known about how these behaviours develop in children [5].

These eating problems, including food selectivity, slow eating, neophobia, lack of interest in food, refusal of and/or aversion to foods in one or more food groups and strong food preferences, are covered by the term “feeding difficulties” (FDs), proposed by Kerzner [6] to standardize parental complaints of feeding problems. FDs are complex and difficult to resolve, and referral for specialized care is essential [7].

FDs are very unique and depend on many factors. In the case of young children, the readiness of a child to accept solid foods, acquired oral–motor skills, taste preferences, etc., significantly affect the development of FDs. In the case of mothers, it is important to understand that children are at the learning stage and are only developing their taste preferences and self-regulatory processes and acquiring the skills for solid food intake [8].

Nonresponsive parental styles regarding food [9] and a lower consumption of fruits and vegetables in children are associated with FDs [10]. Regarding nutritional status, there is no clear consensus; a systematic review presented studies that included children with FDs who were underweight and overweight [11]. Despite the prevalence and impact of FDs in childhood, there has been no research synthesis on the factors associated with the early onset of feeding behaviours in young children [5]. Early identification of these factors is essential due to the related caregiver stress and their relationship with neurodevelopmental conditions [12].

These difficulties are common childhood complaints. Knowing the predictive factors that lead mothers to perceive that their children have FDs is fundamental to understanding this interaction. Mothers are most commonly in charge of determining their children’s daily feeding routines. Mothers usually decide what foods are offered, the amount of food that is provided, the timing and context of meals, and who is involved in feeding interactions [13]. Delays in early intervention may result in simple FDs becoming pervasive or resistant to treatment and increase the risk of malnutrition, growth difficulties, childhood obesity and developmental and cognitive delays [14]. Thus, the purpose of this study was to identify the associations of parental feeding styles, body mass index (BMI), and the consumption of fruits, vegetables and processed foods with mothers’ perceptions of FDs in their children.

## Methods

### Study design and sample

An observational, cross-sectional study was carried out in the waiting room of the Emergency Room at Sabará Hospital Infantil, which serves patients with medical or private insurance plans, in the city of São Paulo. The data were collected in the emergency waiting room by the responsible research nutritionist or by properly trained and supervised nutrition interns. Recruitment took place from November 2017 until October 2018.

This study was conducted according to the guidelines laid down in the Declaration of Helsinki, and the guardians of all individuals included in the study provided informed consent after approval by the Ethics Committee of the PENSI Institute of Sabará Hospital Infantil and the Federal University of São Paulo (UNIFESP) under CAAE no. 59055916.0.0000.5567, (registration 1,760,608) was obtained.

Children were eligible to participate in the study if they (a) were 1–6 years and 11 months of age and (b) had an informed consent form signed by at least one of their parents/legal guardians according to Resolution 196/96 of Brazil’s National Health Council. The exclusion criteria included children who had lost weight in the last 7 days and children with genetic diseases or autism spectrum disorders.

The sample was composed of patients aged 1 to 6 years and 11 months; the sample size calculation was carried out in the statistical program GPower 3.1, and based on the statistical test used, binary logistic regression was performed. With a dichotomous dependent variable, a main continuous predictor exhibiting a normal distribution, and a 95% confidence interval and 80% sampling power, we determined the minimum required sample size to be 154 individuals.

After the hospital screening identified the level of priority and urgency of the patients’ care, we invited the parents of children who were classified as having less serious conditions and the ability to wait for medical care to participate. A total of 278 parents were invited to participate in the study. Children who were not eligible and participants with missing/incomplete data on parental feeding style, BMI, the consumption of fruits, vegetables, processed foods and FDs were excluded. Thus, our final sample included 257 mothers and their children (53.7% boys).

Data on the consumption of fruits, vegetables and processed foods and FDs were collected manually through interviews, and only parental feeding styles were self-reported.

### Independent variables

#### Parental feeding styles

The mothers reported their parental feeding styles using the Caregiver’s Feeding Styles Questionnaire (CFSQ) [15]. This questionnaire has been used [16] and validated in the Brazilian population [17]. The CFSQ was used to assess four parenting styles: authoritative, authoritarian, indulgent, and uninvolved. The complete questionnaire includes 39 items that probe beliefs scored on a 5-point scale (never: 1, rarely: 2, sometimes: 3, almost always: 4, and always: 5) [17].

Score calculations for the two dimensions of responsiveness and demandingness were performed, and a sample mean split for each was used to categorize caregivers into one of four feeding styles (authoritative: high demand, high responsiveness; authoritarian: high demand, low responsiveness; indulgent: low demand, high responsiveness; uninvolved: low demand, low responsiveness). Details about the CFSQ have been described previously [15, 16].

#### Body mass index

The children’s anthropometric data, weight (kg) and height (cm), were measured inside one of the hospital’s triage rooms in accordance with the procedures recommended by the World Health Organization (WHO) [18], and the Food and Nutrition Surveillance System was adopted [19]. Subsequently, nutritional status was determined by the BMI (kg/m^2^) for age and classified by the z score, as proposed by the WHO, by using the WHO Anthro and WHO Anthro Plus programs [18]. The children were categorized into normal weight (≥ z score – 2 to ≤ z score + 1) and overweight (z score > + 1) groups.

#### Consumption of fruits, vegetables and processed foods

To verify the food frequency data for the consumption of fruits, vegetables and processed foods [20, 21], the Food Frequency Questionnaire for Children (FFQC) was used [22].

The compilation of data obtained from the FFQC was carried out using the method proposed by Neumann et al. [23] for use in the paediatric population [21]. This method consists of summarizing the consumption frequencies of foods from a group to a single value, called the “summary measured”. Therefore, for the calculation, the FFQC food frequencies were classified into seven categories: never: 0; <1 time/month: 1; 1–3 times/month: 2; 1 time/week: 3; 2–4 times/week: 4; 1 time/day: 5; and ≥ 2 times/day: 6.

The numerator of the “summary measured” was the sum of the frequencies corresponding to the foods consumed by the children in each food group. The denominator corresponded to the maximum number of foods in each food group multiplied by the maximum frequency code [23]. We obtained food consumption frequency data for fruits, vegetables and processed foods as continuous variables, which were categorized into dichotomous variables, and used the low and high consumption categories for values below or above the median, respectively.

### Dependent variable

Based on previous research [9, 24, 25], the presence or absence of FDs was addressed according to the perceptions of the mothers about the problem through the following question: “Do you think your child has FDs?”.

### Covariates

Child sex (boy and girl), child age (months), and parental education level (university education, high school, and elementary school) were considered covariates in the analyses. These covariates were chosen on the basis of previous evidence indicating their influence on FDs [26, 27].

### Statistical analysis

Descriptive statistics included absolute and relative frequencies, means, and standard deviations (SDs). Binary logistic regression was used to estimate the odds ratio (OR) and 95% confidence interval (95% CI) for the association of parental feeding styles, BMI, and the consumption of fruits, vegetables and processed foods with FDs in children. The reference category of the regression’s dependent variable was the mothers’ perceptions of the absence of FDs in their children. The models were adjusted for child sex, child age, and parental education level. A probability level of 5% was used, and all analyses were performed using SPSS V21 software (SPSS, Inc., IBM Corp., Armonk, New York, NY, USA).

## Results

There were no significant differences (*p* > 0.05) in child sex, child age (months), body mass index (kg/m^2^); frequency of consuming fruits, vegetables or processed foods, or parental education level between the groups with and without FDs. The characteristics of the participants are presented in Table 1. A total of 257 participants were included in the study (aged 1 to 6 years and 11 months). Overall, 53.7% of the sample were boys, and the mean age of the children was 43.8 (SDs: 17.6) months. The mean fruit, vegetable and processed food intakes were 0.40 (SDs: 0.12) and 0.25 (SDs: 0.11), respectively. The majority of the children had normal weight (62.6%), followed by overweight/obesity (37.4%). Furthermore, 58.0% and 54.0% of the children ate less than the median intake of fruits, vegetables and processed foods, respectively. A significant proportion of the parents had attained a university education (mother: 88.5%; father: 79.2%). The indulgent parental feeding style was the most common (40.1%), followed by the authoritative (31.1%), authoritarian (23.7%), and uninvolved (5.1%) styles. The prevalence of FDs was 48.2%.

**Table 1:** Main (Mean [SDs] or n [%]) characteristics of the participants

Variables	Total
Child’s sex, n (%) (*n* = 257)	
*Boy*	138 (53.7)
*Girl*	119 (46.3)
Child’s age, month, mean (SD) (*n* = 257)	43.8 (17.6)
Nutritional status, n (%) (*n* = 257)	
*Normal weight*	161 (62.6)
*Overweight / obesity*	96 (37.4)
Consumption of fruits and vegetables, mean (SD) (*n* = 257)	0.40 (0.12)
*Below median*	149 (58.0)
*Above median*	108 (42.0)
Consumption of processed foods, mean (SD) (*n* = 257)	0.25 (0.11)
*Below median*	138 (54.0)
*Above median*	119 (46.0)
Mother’s age, years, mean (SD) (*n* = 218)	35.4 (5.1)
Father’s age, years, mean (SD) (*n* = 218)	38.1 (6.4)
Mother’s education, n (%) (*n* = 253)	
*University education*	224 (88.5)
*High school*	27 (10.7)
*Elementary school*	2 (0.8)
Father’s education, n (%) (*n* = 250)	
*University education*	198 (79.2)
*High school*	49 (19.6)
*Elementary school*	3 (1.2)
Parental feeding style, n (%) (*n* = 257)	
*Authoritative*	80 (31.1)
*Authoritarian*	61 (23.7)
*Indulgent*	103 (40.1)
*Uninvolved*	13 (5.1)
Feeding difficulties, n (%) (*n* = 257)	
*Absence*	133 (51.8)
*Presence*	124 (48.2)

SDs: standard deviations

Table 2 shows the results of the multivariate logistic regression models for the effects of parental feeding styles, body mass index, and the consumption of fruits, vegetables and processed foods on FDs in children, adjusted for the child sex, child age, and parental education level. An indulgent parental feeding style (OR: 4.66; 95% CI: 2.20–9.85), a high BMI (OR: 1.35; 95% CI: 1.09–1.68), and the consumption of processed foods (OR: 5.21; 95% CI: 2.85–9.53) were positively associated with higher odds of the absence of FDs in children. There were no significant associations of authoritarian or uninvolved parental feeding styles and the consumption of fruits or vegetables with FDs in children (Table 2).

**Table 2:** Logistic regression models [OR (95% CI)] showing association between independent variables with feeding difficulties in children

Variables	ϐ	*p*	OR	95%CI
Parental feeding styles (Ref. Authoritative)		0.000		
Authoritarian	-0.34	0.400	0.70	0.31–1.58
Indulgent	1.53	0.000*	4.66	2.20–9.85
Uninvolved	0.54	0.430	1.73	0.44–6.75
BMI by age (z score)	0.30	0.005*	1.35	1.09–1.68
Consumption of fruits and vegetables (Ref. above median)	0.16	0.610	1.17	0.63–2.17
Consumption of processed foods (Ref. above median)	1.65	0.000*	5.21	2.85–9.53
Constant	-1.62	0.000	0.19	

Y = Absence of feeding difficulties in children, according to maternal perception

The models were adjusted for child’s sex, child’s age, and parent’s education level

OR: odds ratio; CI: confidence intervals

## Discussion

The present study examined the associations of parental feeding styles, BMI, and the consumption of fruits, vegetables and processed foods with mothers´ perceptions of FDs in their children. An indulgent parental feeding style, a high BMI and the consumption of processed foods were positively associated with higher odds of the absence of FDs in children.

One of the predictive factors highlighted was being an indulgent parental feeding style, a fact that contradicts the findings of previous studies, which tend to associate fewer problems at the time of feeding with authoritative parents [9, 28, 29]. However, permissiveness here may be associated with mothers noticing FDs signs in their children and possibly reporting that there were no problems present. A possible explanation for these findings may be that mothers with the indulgent feeding style expose their children to fewer healthy foods, such as fruits and vegetables, and to [30–33] and soft drinks [34] and therefore have fewer problems with food acceptance; however, mothers with the authoritative feeding style probably expose their children to healthier foods and thus report more acceptance problems. Therefore, the positive association between an indulgent parental feeding style and the lack of FDs found in the present study may have occurred due to the way the data were collected (we asked the mothers to classify whether their children presented FDs) and the influence of parental style at the time of the response [31, 35].

Indulgent parents have high levels of responsiveness and respond to their children’s needs at mealtime, but they have lower levels of demand and tend not to make demands regarding the quality and quantity of their children’s food [32, 36]. Hoerr et al. [31] indicated that children of parents with an indulgent and uninvolved parenting style have lower consumption of fruits and vegetables and a greater consumption of foods with high energy density.

Frankel et al. [37] also showed that children of parents with an indulgent parenting style have less ability to self-regulate food intake; therefore, they have a greater tendency to become obese. These findings agree with those of Fisher [38], who found greater self-served portions among children with indulgent and authoritative parents. Other studies also cite the association of this style of parenting with higher BMIs in children [39–42].

High consumption of processed foods was also considered a predictor for mothers considering their children to not have FDs, therefore reinforcing what was mentioned above. Mothers who perceive fewer FDs in their children may offer foods that are more easily accepted by other children in their age group. Regarding the consumption of fruits and vegetables, which are not food groups that are easily accepted by children [43], no associations, whether positive or negative, were observed with mothers’ perceptions of FDs in their children, and no associations, neither positive nor negative, were shown with mothers’ perceptions in our study.

A high BMI was also a predictor of the absence of perceived FDs. This association may be due to the recognition of children’s nutritional statuses and the expectation that food consumption is within reality, perceiving them as children who eat well, regardless of what foods they consume. This idea is supported by Park’s study [44], which showed that parents perceive their children as overweight or obese, and as a result of this perception, they place less pressure on their children to eat.

Another possible explanation to be considered in the results presented in this study is that children are not necessarily always fed by their mothers (who responded to the questionnaire in our research) but can also be fed by other caregivers, such as their fathers, their grandparents and/or third-party caregivers. This can be considered a limiting factor in our study and an idea for future research investigating the interaction between the feeding styles of caregivers who have contact with children.

This study has several limitations, as it was a cross-sectional study, which made conclusions about causes and effects impossible, as it included only the descriptions and associations of the findings. FDs were assessed using only one question despite additional questions being used in previous studies [16, 24]. Therefore, a systematic approach to the evaluation and management of FDs in young children is critical for paediatricians in clinical practice. Data on parental feeding styles were collected through a self-report questionnaire; therefore, measurement error likely occurred. Nevertheless, care was taken to prioritize the order of care established by the hospital and to offer play activities for the children. The temporality of the data collection (i.e., 2017–2018) relative to the release of the FDs rate is limited because the COVID-19 pandemic led to changes in dietary habits and lifestyle parameters, which may lead to impaired nutritional status [45]. However, further research needs to be conducted to assess how associated factors affect FDs incidence.

## Conclusion

This study showed that an indulgent parental feeding style, a high BMI and the consumption of processed foods were positively associated with greater odds of the absence of FDs in children. This research supports the multifactorial features involved in children’s FDs. Therefore, there are factors associated with mothers perceiving their children as having or not having FDs.

## Data Availability

The dataset used and analysed during the current study are available from the corresponding author on reasonable request.

## Declarations

### Note

R² (Nagelkerke) = 0.325. X² (6) of the model = 71.698, *p* < 0.000.
